# Discovering themes in biomedical literature using a projection-based algorithm

**DOI:** 10.1186/s12859-018-2240-0

**Published:** 2018-07-16

**Authors:** Lana Yeganova, Sun Kim, Grigory Balasanov, W. John Wilbur

**Affiliations:** National Center for Biotechnology Information, National Library of Medicine, National Institutes of Health, Bethesda, USA

**Keywords:** Theme discovery, First singular vector, Projection algorithm

## Abstract

**Background:**

The need to organize any large document collection in a manner that facilitates human comprehension has become crucial with the increasing volume of information available. Two common approaches to provide a broad overview of the information space are document clustering and topic modeling. Clustering aims to group documents or terms into meaningful clusters. Topic modeling, on the other hand, focuses on finding coherent keywords for describing topics appearing in a set of documents. In addition, there have been efforts for clustering documents and finding keywords simultaneously.

**Results:**

We present an algorithm to analyze document collections that is based on a notion of a theme, defined as a dual representation based on a set of documents and key terms. In this work, a novel vector space mechanism is proposed for computing themes. Starting with a single document, the theme algorithm treats terms and documents as explicit components, and iteratively uses each representation to refine the other until the theme is detected. The method heavily relies on an optimization routine that we refer to as the projection algorithm which, under specific conditions, is guaranteed to converge to the first singular vector of a data matrix. We apply our algorithm to a collection of about sixty thousand PubMed ^*Ⓡ*^ documents examining the subject of Single Nucleotide Polymorphism, evaluate the results and show the effectiveness and scalability of the proposed method.

**Conclusions:**

This study presents a contribution on theoretical and algorithmic levels, as well as demonstrates the feasibility of the method for large scale applications. The evaluation of our system on benchmark datasets demonstrates that our method compares favorably with the current state-of-the-art methods in computing clusters of documents with coherent topic terms.

**Electronic supplementary material:**

The online version of this article (10.1186/s12859-018-2240-0) contains supplementary material, which is available to authorized users.

## Background

The need for human comprehension of any large document collection has resulted in a plethora of methods aimed at summarizing the collection content. Topic modeling and document clustering are the two most extensively studied directions. Probabilistic topic models, such as Latent Dirichlet Allocation [1], on one hand, summarize a large collection through discovering the latent semantics represented as topical groupings of terms. Clustering methods [2–5], on the other hand, summarize the contents by finding groups of semantically related documents, words or phrases. We, however, believe that topic terms and corresponding document clusters should be integrated at learning time – good term groups provide means to discover good document clusters and vice versa. This is the principal idea behind the thematic analysis methods [6], co-clustering [7, 8] and other approaches that propose document clustering and feature extraction at the same time [9].

Our work is based on the notion of a theme [6], which defines a subject with two equally important representations: a set of documents that discuss a subject, and a set of key terms summarizing the contents of the documents. In an earlier study [6], a theme is computed using a Bayesian framework, which given an initial seed document attempts to find the most probable set of documents discussing the subject of the seed document, and the set of terms which are used to describe that subject. The Expectation maximization algorithm is applied to maximize the likelihood of the database partition into theme and off-theme documents. A similar notion of a theme has further been used in [10–13]. While our approach is inspired by the same dual term- and document-based representations of themes, the mechanism of computing a theme is quite different and is based on a vector space paradigm.

Numerous studies have attempted to use topic modeling and clustering sequentially. LDA topics, for example, are frequently extended to produce topic-based clustering by assigning a document to its highest probability topic and the results have been demonstrated to be a quite strong baseline [14, 15]. Others have explored using topic models to project documents into a topic space prior to clustering [14, 16]. In particular, spectral clustering techniques use the eigenvalues of a similarity matrix of data to perform dimensionality reduction before clustering in fewer dimensions [17]. In addition, document clustering has been derived from the nonnegative matrix factorization [18] and feature extraction methods such as SVD-based latent semantic indexing [19]. Two models that combine document clustering and topic modeling are the clustering topic model, CTM [20], and multigrain clustering topic model, MGCTM [15], which rely on a topic modeling framework that can suffer from the issue of scalability. All of these approaches require simultaneous processing of all topics found.

We first introduce the *projection algorithm*, which given a set of *m* documents and an initial term vector, converges to the optimal term vector that *best* (in the sense of squared projections) represents these *m* documents. We refer to that vector as the *consensus vector*. We then extend the projection algorithm to the *theme algorithm* which detects a theme through an iterative process as follows: it cycles through steps in computing the consensus vector and refining the document set until the theme becomes stable. At every iteration when refining the document set, all documents in the large collection are scored against the current term vector and the top scoring *m* documents are chosen for the next update. Upon convergence we have the document set and the term vector representation which provides a natural summary of the subject. And finally, we demonstrate how one can apply the theme algorithm to find themes in a large document collection.

This study contributes on several dimensions. The projection algorithm represents a theoretical contribution which describes an iterative method to find the first singular vector of the data matrix. We prove the convergence of the algorithm and establish the link between our approach and the power iteration method [21, 22]. We furthermore show that conditions under which the method is guaranteed to converge to the first singular vector are satisfied for our application. In terms of algorithmic contribution, we present the theme algorithm, an approach that starting with a single document detects a theme in a document collection. The theme algorithm is an extension of the projection algorithm, with the difference that it iterates between updating the term vector and the document set based on the updated vector. The projection algorithm is a novel approach to power iteration and provides novel insights. The theme algorithm is novel in that it uses the projection algorithm interleaved with document set updating. We demonstrate the feasibility of the method for large scale applications. The method is scalable and natural to parallelize due to the fact that it computes each theme independently. It is important to note that the method does not depend on the initialization of clusters and yields a unique set of themes.

## Methods

### Projection algorithm

Let *H*^*n*^ denote an *n*-dimensional Hilbert space with inner product (,) and let $\{u_{i}\}^{m}_{i=1}$ denote a finite nonempty set of elements from *H*^*n*^. We are interested in finding a vector *ϕ* that maximizes the sum of squares of projections of all elements in $\{u_{i}\}^{m}_{i=1}$ onto *ϕ*

1 $$\begin{array}{@{}rcl@{}} \phi = \underset{\|\phi'\|=1}{\text{argmax}} \sum\limits_{i} (u_{i},\phi')^{2}  \end{array} $$

This is what we refer to as the *projection problem*. Our interest is in the solution of this problem and its application to exploratory topic analysis.

We begin with the observation that

2 $$\begin{array}{@{}rcl@{}} \sum\limits_{i} \| u_{i} - (u_{i},\phi)\phi \|^{2} = \sum\limits_{i} \left\{ \| u_{i} \|^{2} - (u_{i},\phi)^{2} \right\}  \end{array} $$

so an equivalent statement of the projection problem is

3 $$\begin{array}{@{}rcl@{}} \phi = \underset{\|\phi'\|^{2}=1}{\text{argmin}} \sum\limits_{i} \| u_{i} - (u_{i},\phi')\phi' \|^{2}  \end{array} $$

We define an iterative method which starts with an initial value of *ϕ*_0_ and iterates until an optimal value of *ϕ* for a group of documents $\{u_{i}\}^{m}_{i=1}$ is found.


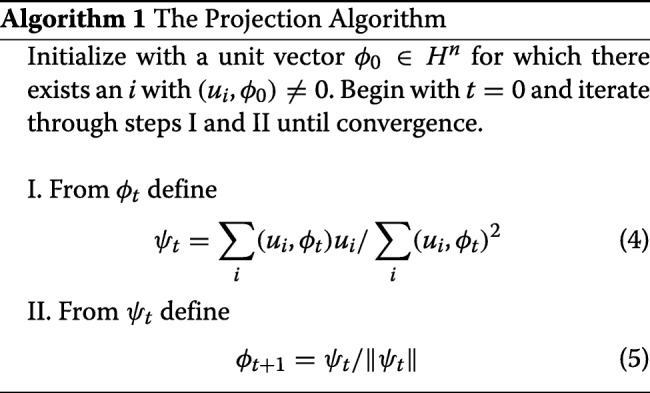


In other words, given a set of *m* documents and an initial term vector, the *projection algorithm* converges to the optimal term vector. We will refer to this vector as the *consensus vector*. In Additional file 1: Analysis of the projection algorithm, we provide the proof of convergence, and identify a convenient stopping criterion for the projection algorithm. We also describe the connection between the projection algorithm and the power iteration method, and provide conditions that guarantee the convergence of the projection algorithm to the first singular vector of the data matrix.

### Projection-based theme discovery

To effectively apply the *projection algorithm* for discovering a theme in a document collection, we modify the algorithm by iteratively updating the set of documents $\{u_{i}^{t}\}_{i=1}^{m}$ along with *ϕ*_*t*_. We refer to this modified algorithm as the theme algorithm (Algorithm 2). At every step *t*, this algorithm updates *ϕ*_*t*_ as well as the set of documents $\left \{u_{i}^{t}\right \}_{i=1}^{m}$, by scoring all the documents in the larger collection against the current term vector and choosing the top scoring *m* documents for the next update. This, in turn, results in a better update for *ϕ*_*t*+1_, etc. The theme algorithm will converge because document set updates will be limited and eventually the algorithm will work with a stable set of documents and become simply the projection algorithm on those documents.


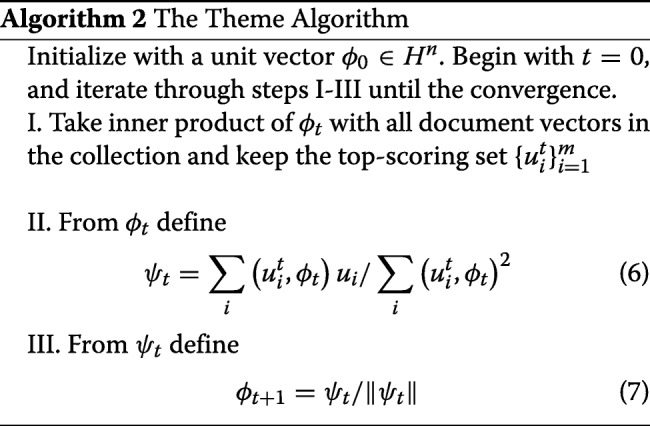


**Corollary (ideal case).** Suppose *V*⊆*S* and |*V*|≥*m*. Further suppose that for any *ϕ*_1_∈*V* and *ϕ*_2_∈*V* and *ρ*∈*S*−*V*, (*ϕ*_1_,*ϕ*_2_)>(*ϕ*_1_,*ρ*). Then, if we choose *ϕ*_0_∈*V*, the algorithm generates a theme contained in *V*.

The choice of *m* is important in the theme algorithm. If we try to imagine the landscape of themes, there would be some very large peaks and a huge number of smaller peaks corresponding to smaller subjects or different facets of larger subjects. We observed that setting large *m* will steer the algorithm into climbing a larger peak and may frequently shift the topic to greater generality. With smaller *m* we are localizing our algorithm to find the peak in a vicinity of the original document. In the language of the corollary, suppose that there is a natural theme *V* and we wish to find it. We would start by choosing *ϕ*_0_∈*V*. If *m*≤|*V*| we can expect the first set of documents in Step 1 to be in *V*. Depending on how closely the assumptions of the corollary hold, we may expect to find a theme that is contained within *V*, whereas if *m*>|*V*| we have no guarantees. To investigate these observations, we perform a series of experiments (discussed in the Experiments and Results section) and examine how topic performance measures change depending on the value of parameter *m*. Based on our observation, we believe setting *m* to 10 provides enough information to define a meaningful term-vector, while keeping a theme focused.

With convergence of the theme algorithm we obtain a consensus vector and scores for all documents against that vector. While only the *m* top scoring documents formally belong to the theme, one can be flexible about number of documents to associate with a theme. The top *m* documents are determined by the theme algorithm. However, some themes are stronger than others and the consensus vector produces many more high scoring documents. We choose to include all the documents scoring half as high as the top scoring document in a theme.

To apply this approach to a large collection, we run the theme algorithm starting with every document in the collection as a seed. All documents are converted to a bag-of-words representation and thence to *tf-idf* vectors [23, 24]. Each seed document has its *tf-idf* vector normalized and used as the *ϕ*_0_ to provide the starting point of the theme algorithm. When all themes have been computed a post processing step is used to remove redundant themes. The computed themes are first sorted by size from the largest to the smallest. Starting at the top of the list, themes that have half or more document overlap with a larger one are dropped.

Our approach produces many themes and we propose the following practical strategy for searching and browsing them by subject areas. Treating each theme as a document, makes them accessible through Boolean querying much as for documents. Because the terms in a theme are weighted by their importance in the theme, these values may be used to rank themes for a given term. Therefore, one can browse the themes that are retrieved in response to a query term in order of their importance to the term and explore the contents of themes by clicking a theme link, which leads to display of the documents in their order of importance to the theme.

In the next section we illustrate our approach by applying it to a subset of PubMed documents examining the subject of Single Nucleotide Polymorphism (SNP). We also present a demo interface, https://www.ncbi.nlm.nih.gov/CBBresearch/Wilbur/IRET/SNP, that allows one to access themes by a query, and from there browse the themes that are retrieved.

### Application to the SNP literature and analysis

A Single Nucleotide Polymorphism is a DNA sequence variation occurring commonly within a population in which a single nucleotide in the genome differs between members of a biological species or paired chromosomes. Variations in the DNA sequences of humans can affect how humans develop diseases and how humans respond to pathogens, drugs, vaccines, and other agents. SNPs are a highly researched topic as they are of great importance in biomedical research for comparing regions of the genome in genome-wide association studies as well as for personalized medicine. Thus identifying various topics discussed in these documents may be of benefit. As of August 2014, the PubMed query ‘single nucleotide polymorphism’ retrieved 63,147 citations, of which 59,046 have both title and abstract. We refer to this dataset of 59,046 documents as the SNP collection and explore it with the goal of finding themes.

Our theme detection methodology is applied starting with each document in the SNP collection as a seed. As described above, each seed document’s vector representative is normalized and provides a starting point for the theme algorithm. We then apply the postprocessing step to remove redundancy. That leaves us with 1066 themes of which 17 contain 200 or more documents, 45 contain between 100 and 200 documents, and the remaining ones have between 20 and 100 documents, and an additional long tail of 5013 smaller themes (between 10 and 20 documents), which we decided not to include in the analysis. Some of the largest topics are on breast cancer, amyotrophic lateral sclerosis, and vascular endothelial growth factor. Table 1 presents the ten largest themes found in the SNP dataset along with the top scoring 10 terms that represent each theme. We have created a web interface (https://www.ncbi.nlm.nih.gov/CBBresearch/Wilbur/IRET/SNP) where one can explore the themes given a query term. In response to a query, the system retrieves themes ranked by the importance of query terms in them. Each theme is presented to the user represented by its top 5 scoring terms.

**Table 1:** Top scoring *Theme*-generated terms for the largest 10 themes in the SNP dataset

Theme size	Top 10 terms
765	breast / breast cancer / cancer / cancer risk / breast neoplasms, genetics/ risk / breast cancer / breast neoplasms / women / controls
438	sle / lupus / lupus erythematosus, systemic / systemic lupus / lupus erythematosus / erythematosus / systemic / sle patients / patients / susceptibility
437	prostate / prostate cancer / cancer / prostatic neoplasms, genetics / prostatic neoplasms / risk / cancer risk / men / p / associated
436	ra / rheumatoid / rheumatoid arthritis / arthritis / arthritis, rheumatoid / arthritis, rheumatoid, genetics / ra patients / controls / susceptibility / association
399	cad / coronary / coronary artery / artery disease / artery / coronary artery disease, genetics / disease cad / coronary artery disease / risk
351	lung cancer / lung / cancer / lung neoplasms / lung neoplasms, genetics / risk / cancer risk / ci / smoking
340	meta analysis / meta / cancer / cancer risk / studies / analysis / polymorphism / model / association / control studies
339	ad / alzheimer’s / alzheimer disease, genetics / alzheimer disease / disease / onset / risk / late onset / aged / ad patients
315	amd / age related / macular/ macular degeneration/ degeneration / macular degeneration, genetics / cfh / age / complement factor / factor h
294	colorectal / colorectal cancer / crc/ cancer / colorectal neoplasms, genetics / colorectal neoplasms / risk / ci / cancer risk / controls

Clusters computed by the theme algorithm provide non-trivial groupings of documents which may be of interest to researchers and clinicians not only by providing a summary view of the literature, but also by bringing to light some associations that are not widely known and can be further explored.

Here we present two examples within the SNP dataset where interesting associations are found as themes.

**FOXP2**: Forkhead box protein P2 (FOXP2) is a protein that, in humans, is encoded by the FOXP2 gene, and is required for proper development of speech and language. Querying the system with ‘foxp2’ retrieves ten themes. In addition to well-known associations, computed themes reveal potential association between FOXP2 and schizophrenia, as well as autism, dyslexia, and, possibly, Asperger syndrome. For example, PMID 20649982 in the top theme describes an association between the FOXP2 gene and language impairment in schizophrenia.

**Sickle Cell Disease**: Querying the system with phrase ‘sickle cell’ retrieves twenty eight themes. The top two themes discuss a well-known association between sickle cell disease and sickle cell anaemia (SCA) with the Klotho gene. The next theme discusses an acute chest syndrome, which is also a known sickle cell disease related complication. Additional themes discuss SCA in the context of malaria, describing how despite the disease’s lethal symptoms, the mutation protects its carriers from malaria. There is also a theme describing the relation between the disease and morphine pharmacokinetics, such as PMID 19357842.

This approach is scalable because it computes the themes independently of each other (i.e. the overall process can be parallelized for efficiency), and uses a greedy method for pruning themes.

## Results and discussion

Evaluating the performance of topic modeling or clustering algorithms is a challenging task. It is challenging not only because manually created gold standards are required, but also because creating such gold standards is not a well-defined task. Results may vary depending on the goal of the task, and be equally useful for their particular tasks. Because our model combines term- and document-based representations, we evaluate our model based on its document clustering performance as well as its ability to compute meaningful topic terms.

### Datasets

The experiments are conducted on the SNP dataset introduced in this paper and the 20-Newsgroups benchmark dataset. The 20-Newsgroups dataset (20NG) is a set of 18,828 messages collected from 20 different Usenet newsgroups (http://people.csail.mit.edu/jrennie/20Newsgroups). We preprocess it by removing stop words and represent each document as a *tf-idf* vector for application of the theme algorithm.

### Evaluating topic-term association with topic coherence measures

Topic coherence measures score a topic by measuring the degree of semantic similarity between high scoring words in the topic. These measures capture the semantic interpretability of the topic based on topic subject terms.

Recent studies have investigated several topic coherence measures in terms of their correlation with human ratings [25, 26]. Two measures that have been demonstrated to correspond well to human coherence judgements are NPMI (normalized point-wise mutual information, also referred to as the UCI measure [27]), and the UMass measure [28]. NPMI is defined as

8 $$\begin{array}{*{20}l} \text{NPMI} = \sum\limits_{k=2}^{K} \sum\limits_{l=1}^{k-1} \frac{\log \frac{p(t_{i},t_{j})+eps}{p(t_{i})p(t_{j})}}{-\log(p(t_{i},t_{j})+eps)},  \end{array} $$

where *p*(*t*_*i*_,*t*_*j*_) is the fraction of documents containing both terms *t*_*i*_ and *t*_*j*_, and *K* indicates the number of top subject terms; *eps*=1/*N* is the smoothing factor, where *N* is the size of the dataset.

The UMass measure defines the score to be based on document co-occurrence counts:

9 $$\begin{array}{*{20}l} \text{UMass} = \sum\limits_{k=2}^{K} \sum\limits_{l=1}^{k-1} \log \frac{D(t_{k},t_{l})+eps}{D(t_{k})},  \end{array} $$

where *D*(*t*_*i*_) is the document frequency of term *t*_*i*_ (the number of documents with at least one token of type *t*_*i*_) and *D*(*t*_*i*_,*t*_*j*_) is the co-occurrence frequency of terms *t*_*i*_ and *t*_*j*_ (the number of documents containing both *t*_*i*_ and *t*_*j*_). As in the NPMI measure, *K* is the number of top terms and *eps*=1/*N* is a smoothing factor included to avoid taking the logarithm of zero. Intuitively, this metric computes the conditional probability of each word given the higher ranked word in the topic.

Here we use the NPMI and the UMass coherence measures to evaluate the topic coherence on the SNP dataset. As mentioned in the previous section, our algorithm applied to the SNP dataset results in 1066 topics of size twenty or more. We evaluated our top scoring terms and compared the results with those computed by LDA. The Mallet open-source tool [29] was used to run LDA on the SNP dataset using unigrams and default parameters. Guided by the number of topics obtained by our method we ran LDA with 1000 topics, and compared the results with the 1066 themes. We also ran LDA with 100 topics, and compared the results with the largest 100 themes computed by *Theme*.

Tables 2 and 3 present the results based on UMass and NPMI coherence metrics respectively for the top 5, 10, and 20 topic words (unigrams) produced by LDA and the *Theme* consensus vectors. *Theme* computations are based on unigrams, bigrams, and MeSH terms and resultant consensus term vectors do include bigrams and MeSH terms in addition to unigrams. For comparison purposes, the evaluation is based on only the top scorings single terms found by *Theme*. In addition, we ran *Theme*_*uni*_, a variant of our algorithm that uses single terms only to compute the themes. *Theme*_*uni*_ generates 1,623 clusters of size twenty or more.

**Table 2:** Comparative evaluation of *Theme*-generated terms with LDA using the UMass coherence metric on the SNP dataset

# Cl	Method	Topic terms		
		Top 5	Top 10	Top 20
100	LDA	-21.15	-108.99	-541.92
100	*Theme*_*uni*_	-15.64	-81.73	-378.95
100	*Theme*	-13.53	-80.12	-397.23
1000	LDA	-33.66	-181.19	-942.08
1623	*Theme*_*uni*_	-19.43	-98.31	-461.09
1066	*Theme*	-17.25	-94.82	-462.35

**Table 3:** Comparative evaluation of *Theme*-generated terms with LDA using the NPMI coherence metric on the SNP dataset

# Cl	Method	Topic terms		
		Top 5	Top 10	Top 20
100	LDA	2.52	8.47	29.33
100	*Theme*_*uni*_	3.34	9.78	27.38
100	*Theme*	3.83	10.38	28.02
1000	LDA	3.04	11.46	44.70
1623	*Theme*_*uni*_	3.34	10.72	30.91
1066	*Theme*	3.80	11.80	33.21

Results demonstrate that top scoring terms computed by both *Theme* and *Theme*_*uni*_ achieve a better coherence score than those computed by LDA for the UMass coherence measure. For the NPMI coherence measure, results are split. *Theme* gives better scores for the top five terms, results are mixed for the top ten, and LDA scores are better for top twenty terms. We also observe that *Theme* produces more coherent clusters than the *Theme*_*uni*_ variation of the algorithm, indicating that bigrams and MeSH Terms provide valuable information.

**Fig. 1:**
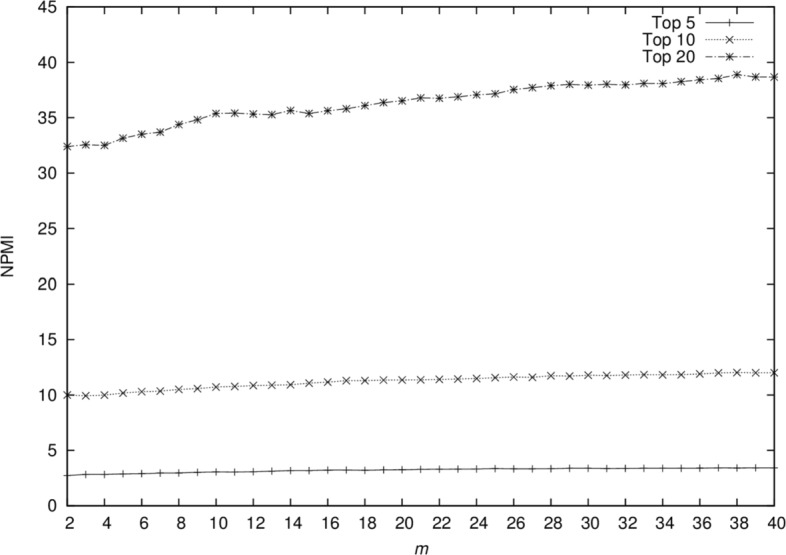
NPMI of top 5, 10, and 20 topic terms. The size of *m* is varied from 2 to 40 and for every value of *m* we compute the NPMI scores for top 5, 10 and 20 terms. We observe that as the size of *m* increases, the coherence of the top terms also increases

**Fig. 2:**
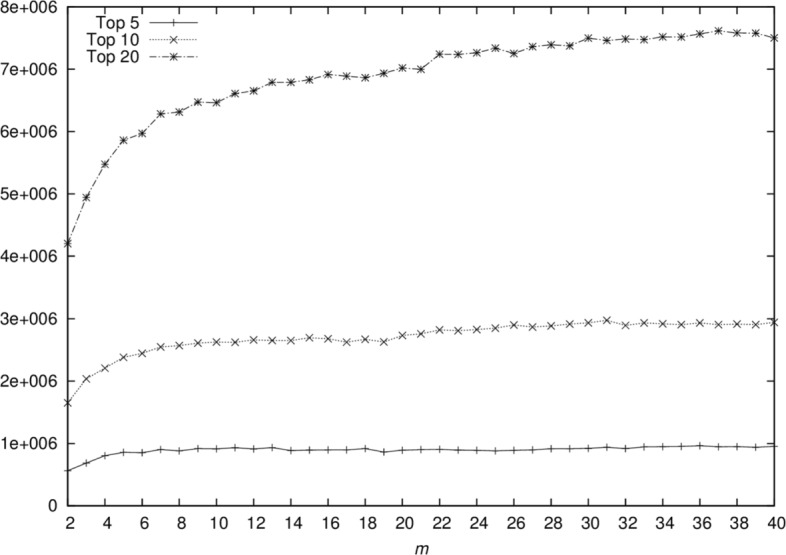
Frequency of top 5, 10, and 20 topic terms. The size of *m* is varied from 2 to 40 and for every value of *m* we compute the average frequency of the top 5, 10 and 20 subject terms. We observe that as the size of *m* increases, the frequency of the top terms also increases, suggesting that the algorithm converges to a more general theme

To understand the factors affecting the NPMI measure in theme generation, we computed NPMI scores for top 5, 10, and 20 terms while varying the size of *m* from 2 to 40. Figure 1 shows that as the size of *m* increases, the coherence of the top terms also increases. We, however, observe that the average frequency of these top subject terms also increases (Fig. 2), suggesting that the algorithm converges to a more general theme for a larger *m*. In an attempt to find a balance between specificity and highly coherent topics, we set *m* to 10, based on empirical observations. Clearly this comes at a cost of lower NPMI coherence for higher numbers of terms.

### Evaluating clustering performance

Working with biomedical literature in PubMed allows us to leverage the availability of the MeSH resource and compute the standard recall-precision values for clustering performance evaluation. MeSH is a controlled vocabulary for indexing and searching biomedical literature [30]. MeSH terms are manually assigned to PubMed articles and are indicative of the main subject of an article. Therefore, these terms can be used to evaluate how well the documents are grouped by topics. For each cluster in the SNP dataset, MeSH terms assigned to papers in the cluster are collected, and p-values of these MeSH terms are calculated using the hypergeometric distribution [31]. Then the average recall and precision values are computed over the three most significant MeSH terms in each cluster and further these are averaged over all clusters. This evaluation technique has been successfully utilized in multiple recent studies in the biomedical domain [13, 32].

We will use this approach to evaluate clustering performance of our algorithm on the SNP dataset and to compare it to LDA-based clustering. The document-topic associations in LDA are computed by coupling a document with the highest probability topic in the document-topic distribution, and is referred to as LDA-Naïve. Previous studies have demonstrated LDA-Naïve to be a rather strong baseline.

Following the setup in the previous experiments LDA-Naïve clusters are generated based on LDA runs with two options for the number of topics, 100 and 1000. To make the comparison between our method and LDA fair in terms of clustering performance, we evaluate the results based on two plausible thresholds. First, we pick the largest one hundred themes produced by our method and compare it with the LDA-Naïve with 100 topics. Second, we extract LDA-Naïve clusters that contain twenty or more documents (587) and compare them with same number of largest clusters found by *Theme*_*uni*_ as presented in Table 4. Precision (P), Recall (R) and F-score (F) are computed and averaged over the number of clusters in each experiment and are presented in Table 4. Since the evaluation is based on MeSH Terms we have to compare LDA-Naïve to the *Theme*_*uni*_ variant of the algorithm, and not the *Theme* variant, because only single words are used to learn the term weights in *Theme*_*uni*_.

**Table 4:** Comparative evaluation of *Theme* and LDA-Naïve clusters on the SNP dataset using precision (P), recall (R), and F-score (F) metrics

# Cl	Method	P	R	F
100	LDA-Naïve-100	0.358	0.364	0.361
100	*Theme*_*uni*_	0.688	0.302	0.419
587	LDA-Naïve-1000	0.507	0.278	0.359
587	*Theme*_*uni*_	0.639	0.226	0.334

Results in Table 4 indicate that clusters computed by LDA-Naïve and *Theme*_*uni*_ are comparable in terms of average F-scores. Clusters computed by *Theme*_*uni*_ are more precise, which is beneficial for our application as given a very large number of documents users usually will only consider the top few documents.

The next series of experiments is performed on the 20NG collection, which is the most widely used benchmark dataset for evaluating clustering performance. Following [33] and [15], we use normalized mutual information (NMI) and accuracy (AC) to measure the clustering performance. Let *C* denote the set of reference clusters and *C*^′^ denote the set of clusters computed by the algorithm. The mutual information is defined as:

10 $$\begin{array}{*{20}l} MI(C,C') = \sum\limits_{c_{i} \in C, c'_{j} \in C'} p(c_{i},c'_{j}) \log_{2} \frac{p(c_{i},c'_{j})}{p(c_{i})p(c'_{j})}  \end{array} $$

and we use the normalized mutual information

11 $$\begin{array}{*{20}l} NMI(C,C') = \frac{MI(C,C')}{\max(H(C),H(C'))},  \end{array} $$

where *H*(*C*) and *H*(*C*^′^) are entropies of *C* and *C*^′^ respectively. For more details please refer to [33].

Accuracy is defined as

12 $$\begin{array}{*{20}l} AC(C,C') = \frac{{\sum\nolimits}_{i} \max_{j} | c_{i} \cap c'_{j} |}{N},  \end{array} $$

where *N* is the total number of documents, *c*_*i*_ is the set of documents in a cluster and $c^{\prime }_{j}$ is the set of documents in a reference cluster.

The *Theme* algorithm is not intended as a flat partitioning method, and neither has it the ability to control the number of clusters to be computed. In order to compare with LDA on 20NG, we apply a greedy method for partitioning the database into exactly 20 clusters based on themes. Every document has a score associated with every theme, which reflects its relevance to the theme. Given any set of themes, we affiliate a document with that theme where it achieves the highest score. Based on these scores, we first select the theme that has the highest sum of scores (ties will be randomly broken). Now we continue our greedy process by adding the theme which maximizes the increment in affiliated scores over all documents. Continue the process until 20 themes are selected and the result is a partition of the database into 20 clusters. As shown in Table 5, our method has an advantage in terms of accuracy and F-score, which comes at a cost of lower NMI.

**Table 5:** Comparative evaluation of *Theme*-generated clusters with LDA-Naïve on the 20NG collection using accuracy (AC), NMI and F-score (F) metrics

Method	AC	NMI	F
*Theme*_*uni*_	53.52	47.98	52.46
LDA-Naïve	50.24	51.50	50.46

### Contrast between LDA topics and Themes

There are important differences between LDA and the *Theme* algorithm. The *Theme* algorithm is based on the *tf-idf* weighting paradigm that has proved so successful in information retrieval [34, 35]. The vectors representing documents are so constructed that the dot product of vectors representing two documents is the sum of the *tf-idf* weights of the words they have in common. Thus, if one of these documents is thought of as a query, the dot product is the score that would be assigned to the other document to determine its ranking to retrieve the most relevant documents in the database. In fact, the related documents in PubMed are determined as the top scoring documents from such dot products. For this purpose, we use a *tf-idf* formulation that has proven most successful in PubMed [23, 24]. Since the theme vector is a weighted sum of the document vectors for those documents representing the theme, it is evident that the theme vector represents a kind of summary of the documents representing the theme on the one hand, while the documents at the same time satisfy the condition that they are the best answers (highest scoring) to the theme thought of as a query.

By contrast LDA is not based on an information retrieval paradigm, but rather on a probabilistic model for document generation whereby documents are conceived to have arisen by random selection of words from topics which are themselves randomly grouped to form the sources of different documents. In LDA clustering, two documents may be assigned the same cluster if they have the same most probable source topic even though this may ignore the majority of words in the documents. Again, topics are not restricted in the number of documents to which they contribute and this tends to make the higher frequency terms more probable than the lower frequency terms. In theme generation this effect is countered by the small number of documents used to generate a theme and the IDF weighting that upweights the lower frequency terms. Because of these differences, themes tend to focus on lower frequency terminology and the documents in themes tend to be more closely related to each other when compared to LDA topic based clusters.

**Fig. 3:**
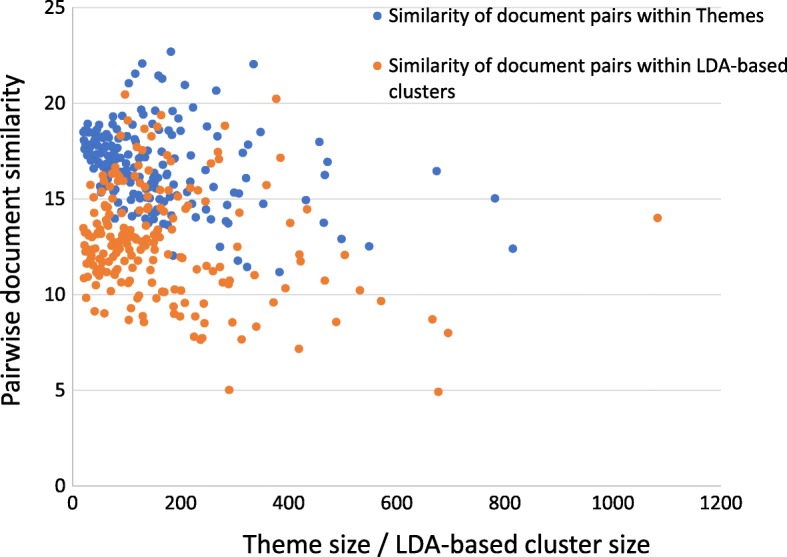
Similarity of document pairs within Themes and LDA-based clusters. The similarity between a pair of documents is computed as the dot product of two document vectors. These values are averaged over all within-theme document pairs and, further averaged over all themes of the same size. Same computation is applied to LDA-based clusters. Each point on the graph presents that average as a function of Themes / LDA-based cluster size

We further explore the differences between these two methods by analyzing the similarity of document pairs within themes and within LDA-based clusters. The similarity between two documents is computed as the dot product of two document vectors to represent how close the two documents are semantically. We compute the average document similarity of all pairs of documents within each theme and similarly within each LDA-based cluster and present the results in Fig. 3. It is evident from the figure that pairs of documents within themes have higher average similarity scores indicating that they are more closely related to each other than document pairs within LDA topics. Furthermore, the overall average similarity of the within-theme document pairs is 16.04, which is considerable higher than the average similarity of the document pairs within LDA based clusters at 9.89. We believe it is then not surprising that themes give a quite different picture of a document collection than do topic based LDA clusters.

**Fig. 4:**
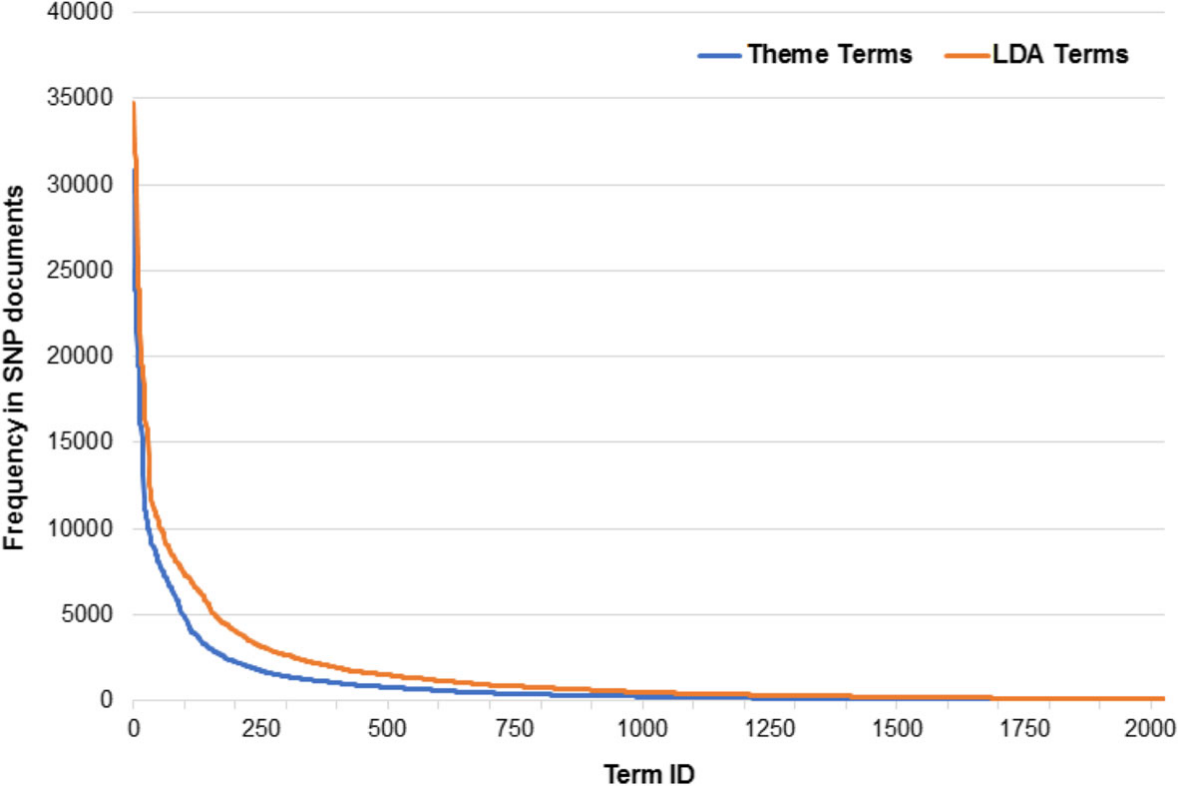
Frequency of *Theme*-generated terms vs. LDA terms. The frequency of *Theme* terms and LDA topic terms in the SNP literature. *Theme*-generated terms are presented in blue, and LDA topic terms are presented in orange

Here we examine the terms most common among the top five terms in LDA topics and Themes. Table 6 presents a comparison of most frequent LDA topic terms and *Theme*-generated terms among the top five for each method. In Table 6 we show number of topics/themes where these terms appear as well as the frequency (in terms of number of documents containing) of these terms in the SNP dataset. Figure 4 is a global comparison of the frequency of theme terms and LDA terms in the SNP literature. The most common among the top five theme terms are significantly more specific than the most common among the top five LDA topic terms. Moreover, the themes appear to have a greater focus on specific diseases or disorders, whereas the topics display a greater focus on more general terms that appear throughout the data. We believe this is a result of the fact that each theme is generated with a small set (10) of documents which can easily focus on a specific disease or medical problem, whereas, topic generation is limited by no such restriction. The fact that themes are created to reflect the content of whole documents and whole documents often focus on a specific disease or medical problem may also be a factor.

**Table 6:** Comparison of most frequent LDA top five topic terms and top five *Theme*-generated terms

LDA term	Freq. in topics /	Freq. in SNP	*Theme* term	Freq. in themes /	Freq. in SNP
	Freq. in themes			Freq. in topics	
polymorphisms	46/0	32,071	cancer	94/14	8,175
gene	45/0	34,735	risk	47/24	20,363
genetic	42/3	29,383	patients	40/37	21,422
associated	37/0	31,365	diabetes	39/7	3,594
patients	37/40	21,422	schizophrenia	36/4	1,806
study	36/0	32,116	dna	36/21	11,098
association	30/11	30,831	genome-wide	32/5	8,100
disease	29/17	15,968	traits	31/6	4,063
analysis	27/1	23,797	method	28/6	6,551
receptor	25/10	7,511	populations	27/11	7,962
two	24/0	17,683	power	26/1	2,171
risk	24/47	20,363	data	23/19	15,234
results	22/0	31,862	loci	23/4	7,006
p	22/11	25,037	genome	23/5	5,790
dna	21/36	11,098	snps	22/18	23,870
genes	20/14	19,411	repair	21/5	1,388
data	19/23	15,234	sequencing	21/4	6,596
snps	18/22	23,870	disorder	21/6	3,517
polymorphism	17/4	23,162	haplotype	21/5	8,933
cell	16/11	5,832	expression	21/10	9,020

Column 1 lists the most frequent LDA terms, followed by number of LDA topics/themes that contain that term in Column 2, and frequency of the term in the SNP dataset in Column 3. Columns 4-6 present similar information for the most frequent *Theme*-generated terms

### Efficiency and Scalability

To demonstrate the efficiency of our method, we generate themes for a collection of 1,000,000 PubMed documents. These are the most recent 1,000,000 PubMed articles that have an abstract of 100 characters or longer.

Since each theme is computed independently, we distribute the computation of the 1,000,000 initial themes among 100 processes, each targeting 10,000 seeds. The computation is set up on a local cluster machine. As a result, 487,222 seeds converge to themes containing 10 or more documents. The slowest of the 100 processes took 1360 min (22.6 h) to run, while the fastest took 799 min (13.3 h). The average run time over 100 processes was about 18 h, and the variation in time between the slowest and the fastest process was mainly due the variable load of the nodes on a cluster machine. The average time for a single seed to converge to a theme within the computational space of 1 million documents was 6.4 s (average computed over 1 million seeds). The average incremental run time of the algorithm is purely linear. The post processing step is then applied to remove the redundant themes and takes 164 min (2.7 h) to compare 487,222 initial themes, resulting in the final set of 159,676 themes, each containing 10 or more documents.

Under the current settings, a total time spent for computing themes is 25.4 h (22.6 h for computing initial themes, and 2.7 h for post processing). However, since the theme computation is parallelizable, the run time of the algorithm is mainly determined by the computational capacity of the computing system and can be made faster depending on number of computers or threads available. For example, if we set 1000 processes to run in parallel instead of 100, the average processing time for each process would be reduced by a factor of ten and result in the total run time of 5 h. This demonstrates the scalability of the method and its’ feasibility for large datasets.

## Conclusion

In this paper, we present a novel algorithm that finds themes in document collections. We define a theme as a subject area characterized by two components: a set of documents and a set of key terms. Our approach treats terms and documents as explicit elements which iteratively refine each other until the theme is found. The method relies on the Projection algorithm, an optimization routine for efficiently finding the first singular vector, which, intuitively, defines the main subject of a theme. We examine the Projection algorithm and provide conditions under which the algorithm is guaranteed to converge to the first singular vector of a data matrix.

The *Theme* algorithm (*m*=10) starts with a single document and its nearest neighbors and operates in a very narrow space, which makes the theme computation efficient. This leads to themes being quite specific, while topics found by LDA tend to be more general. As we have shown, the tightly focused themes produced by the theme algorithm present a different view of the literature than that provided by LDA topic analysis, a view we believe is useful for exploratory topic browsing in PubMed. Another characteristic that distinguishes our algorithm is scalability because it computes each theme independently, and is trivial to parallelize.

Our immediate plan is applying this analysis to the PubMed database, which currently contains over 27 million citations. While topic modeling has been applied to certain subsets of PubMed, applying it to all of PubMed has not been shown yet.

## Additional file

Additional file 1 Analysis of the projection algorithm. The file provides the proof of convergence, and identifies a convenient stopping criterion for the projection algorithm. (CVS 149 kb)

## Abbreviations

AC: Accuracy
20NG: 20-newsgroups dataset
F: F-score
FOXP2: Forkhead box protein P2
NMI: Normalized mutual information
NPMI: Normalized point-wise mutual information
P: Precision
R: Recall
SCA: Sickle cell anaemia
SNP: Single nucleotide polymorphism

## References

[1] Blei D, Ng A, Jordan M (2003). Latent Dirichlet allocation. J Mach Learn Res.

[2] Aggarwal C, Zhai C (2012). A Survey of Text Clustering Algorithms. Mining Text Data, vol 4.

[3] Anastasiu D, Tagarelli A, Karypis G (2013). Document Clustering: The Next Frontier. Data Clustering: Algorithms and Applications.

[4] Jain A, Murty M, Flynn P (1999). Data clustering: A review. ACM Comput Surv.

[5] Xu R, Wunsch D (2005). Survey of clustering algorithms. IEEE Trans Neural Netw.

[6] Shatkay H, Wilbur WJ. Finding themes in MEDLINE documents: Probabilistic similarity search. In: Proceedings of the IEEE Conference on Advanced Digital Libraries: 2000. p. 183–92.

[7] Dhillon IS. Co-clustering documents and words using bipartite spectral graph partitioning. In: Proceedings of the Seventh ACM SIGKDD International Conference on Knowledge Discovery and Data Mining: 2001. p. 269–74.

[8] Busygin S, Prokopyev O, Pardalos P (2008). Biclustering in data mining. Comput Oper Res.

[9] Frigui H, Nasraoui O. Simultaneous clustering and attribute discrimination. In: Proceedings of the IEEE International Conference on Fuzzy Systems: 2000. p. 158–63.

[10] Shatkay H, Edwards S, Wilbur WJ, Boguski M. Genes, themes and microarrays: using information retrieval for large-scale gene analysis. In: Proceedings of the International Conference on Intelligent Systems for Molecular Biology: 2000. p. 317–28. 10977093

[11] Wilbur WJ. A thematic analysis of the AIDS literature. In: Proceedings of the Pacific Symposium on Biocomputing: 2002. p. 386–97. 11928492

[12] Shatkay H, Edwards S, Wilbur WJ, Boguski M. Applying probabilistic thematic clustering for classification in the TREC 2005 Genomics Track. In: Proceedings of the Text Retrieval Conference: 2005.

[13] Kim S, Wilbur WJ (2012). Thematic clustering of text documents using an EM-based approach. J Biomed Semant.

[14] Lu Y, Mei Q, Zhai C (2011). Investigating task performance of probabilistic topic models: an empirical study of PLSA and LDA. Inf Retr.

[15] Xie P, Xing E. Integrating document clustering and topic modeling. In: Proceedings of Conference on Uncertainty in Artificial Intelligence: 2013. p. 694–703.

[16] Xu W, Gong Y. Document clustering by concept factorization. In: Proceedings of the 27th Annual International ACM SIGIR Conference on Research and Development in Information Retrieval: 2004. p. 202–9.

[17] Ng AY, Jordan MI, Weiss Y. On spectral clustering: Analysis and an algorithm. In: Advances in Neural Information Processing Systems: 2001. p. 849–56.

[18] Xu W, Liu X, Gong Y. Document clustering based on non-negative matrix factorization. In: Proceedings of the 26th Annual International ACM SIGIR Conference on Research and Development in Information Retrieval (SIGIR ’03): 2003. p. 267–73.

[19] Deerwester S, Dumais S, Landauer T, Furnas G, Harshman R. Indexing by latent semantic analysis. J Am Soc Inf Sci. 1990;41(6).

[20] Wallach H. Structured topic models for language. PhD thesis. 2008.

[21] Golub G, Van Loan C (2012). Matrix Computations.

[22] Strang G (2009). Introduction to Linear Algebra.

[23] Kim W, Aronson AR, Wilbur WJ. Automatic MeSH term assignment and quality assessment. In: Proceedings of the AMIA Annual Symposium: 2001. p. 319–23. PMC2243528 11825203

[24] Lin J, Wilbur WJ (2007). PubMed related articles: a probabilistic topic-based model for content similarity. BMC Bioinformatics.

[25] Aletras N, Stevenson M. Evaluating topic coherence using distributional semantics. In: Proceedings of the 10th International Conference on Computational Semantics (IWCS 2013): 2013.

[26] Röder M, Both A, Hinneburg A. Exploring the space of topic coherence measures. In: Proceedings of the Eighth ACM International Conference on Web Search and Data Mining: 2015. p. 399–408.

[27] Newman D, Noh Y, Talley E, Karimi S, Baldwin T. Evaluating topic models for digital libraries. In: Proceedings of the 10th Annual Joint Conference on Digital Libraries (JDCL ’10): 2010. p. 215–24.

[28] Mimno D, Wallach H, Talley E, Leenders M, McCallum A. Optimizing semantic coherence in topic models. In: Proceedings of the Conference on Empirical Methods in Natural Language Processing: 2011. p. 262–72.

[29] McCallum A. MALLET: A Machine Learning for Language Toolkit. 2002. http://mallet.cs.umass.edu.

[30] Lowe HJ, Barnett GO (1994). Understanding and using the medical subject headings (MeSH) vocabulary to perform literature searches. J Am Med Assoc.

[31] Kim W, Wilbur WJ (2001). Corpus-based statistical screening for content-bearing terms. J Am Soc Inf Sci Technol.

[32] Yeganova L, Kim S, Wilbur WJ (2014). Retro: concept-based clustering of biomedical topical sets. Bioinformatics.

[33] Cai D, He X, Han J (2011). Locally consistent concept factorization for document clustering. IEEE Trans Knowl Eng.

[34] Witten IH, Moffat A, Bell TC (1999). Managing Gigabytes (2nd Ed.): Compressing and Indexing Documents and Images.

[35] Robertson S, Zaragoza H (2009). The probabilistic relevance framework: BM25 and beyond. Found Trends Inf Retr.

