# Supplementary material for: Discovering themes in biomedical literature using a projection-based algorithm
Source: BMC Bioinformatics. 2018 Jul 16;19:269. doi: 10.1186/s12859-018-2240-0 (PMC6048865; doi:10.1186/s12859-018-2240-0)
Supplement: Supplementary file 1 — Analysis of the projection algorithm. The file provides the proof of convergence, and identifies a convenient stopping criterion for the projection algorithm. (CVS 149 kb) [file 12859_2018_2240_MOESM1_ESM.cvs]

**Analysis of the projection algorithm**

Presented below are two lemmas that when combined yield the solution of the projection problem.

**Lemma 1**. Let $\left\{v_{i}\right\}_{i=1}^{m}$ be a set of vectors from $H^{n}$ and let $\left\{w_{i}\right\}_{i=1}^{m}$ be a set of nonnegative real numbers with $\sum_{i} w_{i}>0$. Then the unique $x$ that minimizes

|  | $\sum_{i} w_{i}\left\Vert v_{i}-x\right\Vert^{2}$ | (A1) |
| --- | --- | --- |

is given by

|  | $x=\sum_{i} w_{i}v_{i}/\sum_{i} w_{i}$ | (A2) |
| --- | --- | --- |

*Proof*: This corresponds to a simple coordinate wise optimization and the result is confirmed for each coordinate with elementary calculus. *QED.*

**Lemma 2**. Let $\left\{u_{i}\right\}_{i=1}^{m}$ be a set of vectors from $H^{n}$ and let $\varphi\in H^{n}$ satisfy $\left\|\varphi\right\|=1.$ Then the expression

|  | $\sum_{i} \left\Vert u_{i}-a_{i}\varphi\right\Vert^{2}$ | (A3) |
| --- | --- | --- |

is uniquely minimized by the choice

|  | $a_{i}=\left(u_{i},\varphi\right),\text{ }\forall i.$ | (A4) |
| --- | --- | --- |

*Proof*: This result follows from the equation

|  | (A5) |
| --- | --- |

*QED*.

Let be any unit vector in $H^{n}$ and suppose that is not orthogonal to all the $\left\{u_{i}\right\}_{i=1}^{m}$. Consider the expression and the fact that the coefficients of are optimal for minimizing this expression according to Lemma 2. Rewrite

|  | (A6) |  |
| --- | --- | --- |

Define

|  | (A7) |  |
| --- | --- | --- |

Then by applying Lemma 1 we obtain $\psi_{t}$ that minimizes (A6)

|  | (A8) |
| --- | --- |

This concludes the analysis for the first step of the algorithm. Next, we show that and to complete the cycle we normalize it

|  | (A9) |
| --- | --- |

By construction if we must have

|  | (A10) |
| --- | --- |

For this to be true it requires that $\psi_{t}$ and are not parallel to each other. Otherwise we would have and we know from Lemma 2 that

would have to achieve its minimum at c = 1 in contradiction to (A10). Also, from (A8) it is easy to see that

|  | (A11) |
| --- | --- |

and if (A10) is true, we may conclude that and hence we normalize it.

From these arguments it is evident that

|  | (A12) |
| --- | --- |

This proves one step of the algorithm increases the sum of squares of the projections (See equation (1)). In addition, we have a relation on successive as follows:

| $\left\Vert\varphi_{t+1}-\varphi_{t}\right\Vert^{2}\leq\left\Vert\psi_{t}\right\Vert^{2}-1=\left\Vert\psi_{t}-\varphi_{t}\right\Vert^{2}$ | (A13) |
| --- | --- |

This can be proved by direct calculation and making use of (A9) and (A11):

|  | (A14) |
| --- | --- |

The relation on successive provides a practical stopping criterion of the algorithm. It is especially attractive because it is available as the byproduct of the algorithm as the value of $||\psi_{t}||$ is computed at every iteration.

Let $S^{n-1}$ denote the set of unit vectors in $H^{n}.$Since $F\left(\varphi\right)=\sum_{i} \left(u_{i},\varphi\right)^{2}$ is a continuous function on the compact set $S^{n-1}$, there must exist a point $\gamma$ where $F$achieves its maximum.

Clearly this is a fixed point for the projection algorithm and yields

| $\gamma=\sum_{i} \left(u_{i},\gamma\right)u_{i}/\sum_{i} \left(u_{i},\gamma\right)^{2}$ | (A15) |
| --- | --- |

Let A be the $m\text{ x }n$ matrix whose rows are the $\left\{u_{i}\right\}_{i=1}^{m}$. Then simple algebra with Eqn. (A15) shows

| $A^{T}A\gamma=\left(\sum_{i} \left(u_{i},\gamma\right)^{2}\right)\gamma$ | (A16) |
| --- | --- |

so $\gamma$ is a principal eigenvector of the self adjoint matrix $A^{T}A$. In this same manner, any fixed point for the algorithm can be shown to be an eigenvector for the matrix. Any eigenvector for the matrix can be shown to be a fixed point for the algorithm by using the argument about parallels after equation (A10). In fact, it is evident that

the projection algorithm applied to $\left\{u_{i}\right\}_{i=1}^{m}$ exactly carries out the power iteration method applied to the symmetric matrix $A^{T}A$ and has the same convergence rate. Given that we start the projection algorithm with an initial guess that has a nonzero component in the direction of an eigenvector associated with the dominant eigenvalue of $A^{T}A$, the algorithm is guaranteed to converge to that eigenvector.

Let us call the set $\left\{u_{i}\right\}_{i=1}^{m}$oriented if the condition

| $(u_{i},u_{j})\geq0\text{, }\forall i,j\text{ s.t. }1\leq i,j\leq m$ | (A17) |
| --- | --- |

is satisfied. Let us define a graph Γ based on the set $\left\{u_{i}\right\}_{i=1}^{m}$ for which the set of nodes is the set of vectors $u_{i},1\leq i\leq m$, and where an undirected edge exists between two nodes $u_{i}$and $u_{j}$ if and only if $(u_{i},u_{j})\neq0$. We will refer to Γ as the associated graph corresponding to $\left\{u_{i}\right\}_{i=1}^{m}$.

**Theorem 1.** Let ${\{u_{i}\}}_{i=1}^{m}$ be an oriented set whose associated graph is connected. Then there is a unique fixed point $\gamma$ for the projection algorithm that satisfies

|  | $\left( u_{i},\gamma\right)>0,\forall i, 1\leq i\leq m$ | (A18) |
| --- | --- | --- |

Further for any starting point $\phi_{0}$ satisfying

|  | $\left( u_{i},\phi_{0} \right)\geq0,\forall i, 1\leq i\leq m$ | (A19) |
| --- | --- | --- |

the projection algorithm converges to $\gamma$.

*Proof*: Let $\varphi_{0}$ be any starting point for the projection algorithm that satisfies (A19). Note that there are generally multiple such starting points because by (A10) any $u_{i}$ normalized would satisfy this condition. Now we show by induction that

|  | $\left( u_{i},\phi_{t} \right)\geq0,\forall i, 1\leq i\leq m$ | (A20) |
| --- | --- | --- |

for all *t*. This is true by assumption for $t=0$. Suppose it is true for $t$. Then from (A1), (A10), and the induction assumption we have

|  | $\left( u_{i},\psi_{t} \right)\geq0,\forall i, 1\leq i\leq m$ | (A21) |
| --- | --- | --- |

The result then follows from the definition (A2). The identification of the projection algorithm with the power iteration method insures that the sequence ${\{\phi_{t}\}}_{t=1}^{\infty}$ converges to a fixed point $\gamma$ which clearly must also satisfy

|  | $\left( u_{i},\gamma\right)\geq0,\forall i, 1\leq i\leq m$ | (A22) |
| --- | --- | --- |

Let $I$ be the set of indices $i$ for which$\left(u_{i},\gamma\right)>0$ and note that $I$ cannot be empty because $\gamma$ is a nonzero vector in the space spanned by the set $\left\{u_{i}\right\}_{i=1}^{m}$. Likewise, let $J$ denote the set of indices $j$ for which $\left( u_{j},\gamma\right)=0.$ Suppose $J\neq\emptyset$. Then by the connectedness of the associated graph there must exist a pair of indices $i\in I$ and $j\in J$ with $\left(u_{i},u_{j}\right)>0$. But then using the fact that $\gamma$ is a fixed point and (A15) and (A22) we see that

|  | $\left( {\gamma,u}_{j} \right)=\frac{\sum_{k} \left( u_{k},\gamma\right){(u}_{k},u_{j})}{\sum_{k} \left( u_{k},\gamma\right)^{2}}$ $\geq\frac{{(u}_{i},\gamma)(u_{i},u_{j})}{\sum_{k} {(u_{k},\gamma)}^{2}}>0$ | (A23) |
| --- | --- | --- |

This contradiction shows that $J$ must be empty and this $\gamma$ must satisfy (A18).

Our next step is to show that the vector $\gamma$ obtained in this way is unique. Again let $A$ be the $m\times n$ matrix whose rows are the ${\{u_{i}\}}_{i=1}^{m}$ and consider that the element in the $i^{th}$ row and the $j^{th}$ column of the matrix $AA^{T}$ is $(u_{i}{, u}_{j})$. From the fixed point $\gamma$ define the vector

|  | $\eta_{\gamma}=(\left( u_{1},\gamma\right),\left( u_{2},\gamma\right),\ldots,\left( u_{m},\gamma\right)$) | (A24) |
| --- | --- | --- |

Note that it satisfies the relation

|  | $AA^{T}\eta_{\gamma}^{T}=(\sum_{i} {(u_{i},\gamma)}^{2})\eta_{\gamma}^{T}$ | (A25) |
| --- | --- | --- |

Now by assumption $\left\{u_{i}\right\}_{i=1}^{m}$ is oriented and its associated graph is connected and these assumptions translate to imply that $AA^{T}$ is a nonnegative irreducible matrix. For such a matrix the Perron-Frobenius theorem asserts that there is a unique largest eigenvalue and corresponding unique eigenvector with strictly positive components. We may conclude that $\eta_{\gamma}$ is this unique positive eigenvector. Thus, if from some different starting point, say $\phi_{0}^{'}$ satisfying (A19) we apply the projection algorithm and converge to $\gamma'$ we must have the equality $\eta_{\gamma'}=\beta\eta_{\gamma}$ for some $\beta>0$. But we can only have this equality if $\gamma'=\beta\gamma$ because these vectors both lie in the space generated by the set $\left\{u_{i}\right\}_{i=1}^{m}$. Because both vectors are of unit length this implies $\beta=1.$We have established that $\gamma$ is the unique fixed point that satisfies (A18) and that from any starting point satisfying (A19) the projection algorithm must converge to $\gamma$. *QED*.
